# Prognostic value of cardiovascular disease status: the Leiden 85-plus study

**DOI:** 10.1007/s11357-012-9443-5

**Published:** 2012-07-04

**Authors:** Petra G. van Peet, Yvonne M. Drewes, Anton J. M. de Craen, Rudi G. J. Westendorp, Jacobijn Gussekloo, Wouter de Ruijter

**Affiliations:** 1 Department of Public Health and Primary Care, Leiden University Medical Center, Postzone V0-P, P.O. Box 9600, 2300 RC Leiden, The Netherlands; 2 Department of Gerontology and Geriatrics, Leiden University Medical Center, Leiden, The Netherlands; 3 Netherlands Consortium for Healthy Ageing, Leiden, The Netherlands

**Keywords:** Aged 80 and over, Cardiovascular disease, Prevention, Cardiovascular morbidity, Functional status, Mortality

## Abstract

This study aimed to explore the prognosis of very old people depending on their cardiovascular disease (CVD) history. This observational prospective cohort study included 570 participants aged 85 years from the general population with 5-year follow-up for morbidity, functional status, and mortality. At baseline, participants were assigned to three groups: no CVD history, “minor” CVD (angina pectoris, transient ischemic attack, intermittent claudication, and/or heart failure), or “major” CVD (myocardial infarction [MI], stroke, and/or arterial surgery). Follow-up data were collected on MI, stroke, functional status, and cause-specific mortality. The composite endpoint included cardiovascular events (MI or stroke) and cardiovascular mortality. At baseline, 270 (47.4 %) participants had no CVD history, 128 (22.4 %) had minor CVD, and 172 (30.2 %) had major CVD. Compared to the no CVD history group, the risk of the composite endpoint increased from 1.6 (95 % confidence interval [CI], 1.1–2.4) for the minor CVD group to 2.7 (95 % CI, 2.0–3.9) for the major CVD group. Similar trends were observed for cardiovascular and all-cause mortality risks. In a direct comparison, the major CVD group had a nearly doubled risk of the composite endpoint (hazard ratio, 1.8; 95 % CI, 1.2–2.7), compared to the minor CVD group. Both minor and major CVD were associated with an accelerated decline in cognitive function and accelerated increase of disability score (all *p* < 0.05), albeit most pronounced in participants with major CVD. CVD disease status in very old age is still of important prognostic value: a history of major CVD (mainly MI or stroke) leads to a nearly doubled risk of poor outcome, including cardiovascular events, functional decline, and mortality, compared with a history of minor CVD.

## Introduction

Cardiovascular disease (CVD) is characterized by a high prevalence and incidence up to the highest age groups. Moreover, cardiovascular morbidity is an important cause of disability and, from middle age onwards, CVD is the leading cause of death (Roger et al. 2011; McDermott 2007). Therefore, prevention of cardiovascular events has high priority and risk prediction models have been developed.

In daily practice, populations are usually dichotomized into people with known atherothrombotic CVD, such as coronary heart disease, stroke/transient ischemic attack (TIA), and peripheral arterial disease, and people without those manifest conditions, but possibly with risk factors for CVD, such as hypertension, hypercholesterolemia, diabetes, or smoking (Wilson et al. 1998; Wilson 2011; Graham et al. 2007; Dornbrook-Lavender et al. 2003). Persons without manifest CVD theoretically qualify for the so-called primary prevention, be it on a population scale or on a more personal level when their calculated CVD risk exceeds predefined thresholds (Graham et al. 2007; Wald et al. 2011). Persons with prior CVD are known to have a high risk of recurrent CVD (Kerr et al. 2009; Vaartjes et al. 2010; Steg et al. 2007; van Wijk et al. 2005) and should, therefore, receive optimal “secondary prevention,” including lifestyle advice and preventive medication.

Despite evidence of its value also in old age, elderly people do not receive optimal preventive treatment even after major events (Arnold et al. 2011; Kvan et al. 2006; Bhatt et al. 2006). At very old age, drug interactions, intoxications, and adverse effects can have serious impact on the quality of life (Banerjee et al. 2011) Therefore, further risk differentiation within those with prior CVD might help clinicians to select those at the highest risk of recurrent events. In younger age groups, it is already known that patients with prior CVD are at the highest risk of a recurrent cardiovascular event (Kerr et al. 2009). Within patients with prior CVD, a recent study showed that a history of ischemic events leads to a greater risk of future events than a history of stable coronary, cerebrovascular, or peripheral artery disease (Bhatt et al. 2010). At present, it is unknown whether these findings can also be applied to patients aged 85 and over.

We hypothesized that subgroups with different risks of recurrent CVD might also be observed within the population of the oldest old. A history of myocardial infarction (MI) or stroke might have a different prognosis than a history of relatively “minor” CVD such as stable angina or claudication, TIA, or milder cases of heart failure. This may have clinical consequences for the format and intensity of secondary prevention in these groups of older people.

We investigated whether differences in prognosis exist between very old people with various levels of prevalent CVD, compared to those with no manifest CVD. Since in older populations the outcomes “morbidity” and “functional status” become even more important than mortality, we studied the prognosis not only regarding (cause-specific) mortality, but also with respect to recurrent CVD morbidity and functional status.

## Methods

### Study population

The Leiden 85-plus Study is a prospective population-based study in 85-year-old inhabitants of the city of Leiden, The Netherlands. The study design and characteristics of the cohort have previously been described in detail (der Wiel et al. 2002; van Exel et al. 2002). In brief, between September 1997 and September 1999, 705 people from the 1912–1914 birth cohorts living in the city of Leiden who reached the age of 85 years were eligible to participate. No exclusion criteria were used. From the 705 people who were eligible at age 85, 92 refused participation and 14 died before enrolment. A total of 599 (87 %) people gave informed consent and were enrolled. At baseline and yearly up to the age of 90 years, participants were visited at their place of residence to obtain extensive data on health, functioning, and well-being. In addition, a medical history was obtained from the participant’s primary care physician. For all participants, classic cardiovascular risk factors were determined. The Medical Ethics Committee of the Leiden University Medical Centre approved the study.

### Prevalence of CVD at age 85 years

For each participant, the primary care physician was interviewed about the history of CVD using a standardized questionnaire, which included questions on present and past cardiovascular pathologies, including MI, stroke, surgery for arterial disease (aorta, carotid, coronary, or peripheral arteries), angina pectoris, TIA, intermittent claudication, and heart failure. An ECG was recorded. The presence of MI on the ECG was defined as the presence of Minnesota Code 1-1 or 1-2 (excluding 1-2-8). Participants were assigned to three different groups according to their CVD status: a group with no known history of CVD (reference group), a group with a history of “minor” CVD, and a group with a history of “major” CVD. Minor CVD was considered present if the primary care physician had recorded a history of angina pectoris, TIA, intermittent claudication, and/or heart failure. Major CVD was defined as a history of MI (including MI on baseline ECG), stroke, or surgery for arterial disease (aorta, carotid, coronary, or peripheral arteries). These criteria for minor and major CVD were based on literature in younger age groups (Bhatt et al. 2006; Rosengren et al. 1998).

### Clinical endpoints

#### (Non)fatal myocardial infarction

Up to 90 years of age, all incident fatal and nonfatal MIs were annually registered using data from the primary care physician, ECGs, and death registration forms. Incident MI on the ECG was defined as the appearance of Minnesota Code 1-1 or 1-2 or Minnesota Code 1-3 in combination with the first appearance of Minnesota Code 5-x in the same myocardial area (Macfarlane and Latif 1996). A fatal incident MI was categorized by cause of death codes I21–I23 (International Classification of Diseases [ICD]-10).

#### (Non)fatal stroke

Information on incident stroke was collected annually from the primary care physician up to 90 years of age. A fatal incident stroke was categorized by cause of death codes I61–I69 (ICD-10).

#### Incident cardiovascular events or cardiovascular mortality

The composite endpoint “incident cardiovascular events or cardiovascular mortality” was defined as fatal and nonfatal MI, fatal and nonfatal stroke, or other cardiovascular mortality.

#### Mortality

All participants were followed up for mortality until the age of 95 years. Dates and causes of death were obtained from civic and national registries. Causes of death were divided into cardiovascular causes (ICD-10 codes I00–I99) and noncardiovascular causes (all other ICD-10 codes). Assignment of cause of death was done blinded for baseline and follow-up study data.

#### Functional status

Up to 90 years of age, participants were annually visited by a research nurse at their place of residence. Cognitive function was assessed by the Mini-Mental State Examination (MMSE) with scores ranging from 0 to 30 points (optimal) (Tombaugh and McIntyre 1992). Disability was assessed using the Activities of Daily Living (ADL) items from the Groningen Activity Restriction Scale with scores ranging from 9 (optimal) to 36 points (Kempen et al. 1996). In those with MMSE scores above 18, Cantril’s Ladder of Life with a score from 1 to 10 (optimal) points was used as a measure of general well-being (Cantril 1965) and the 15-item Geriatric Depression Scale (GDS) with scores ranging from 0 (optimal) to 15 points was used to screen for depressive symptoms (de Craen et al. 2003).

### Statistical analysis

Differences in baseline characteristics between the groups according to CVD status were analyzed with the chi-square test for categorical variables and the Jonckheere–Terpstra test for continuous variables. Time-to-event curves were constructed with the Kaplan–Meier method and compared using a log-rank test. If no exact time to event was available, the time to event was calculated as halfway that particular year. Mortality and morbidity hazard ratios (HR) and corresponding 95 % confidence intervals (CIs) were calculated in a Cox proportional hazards model adjusted for sex. The same HRs were calculated in a model with additional adjustments for the use of antihypertensive medication, income, and level of education. Incidence rate was calculated using the timetable method as number of incidents per 1,000 person-years at risk with corresponding 95 % CIs. Differences in cognitive function (MMSE), changes in disability (ADL), general well-being (Cantril), and depressive symptoms (GDS) were estimated using linear mixed models adjusted for sex and are presented as (predicted) means with standard errors. As a first sensitivity analysis, the stratification in groups according to CVD status was repeated at the age of 90 years, with updated information about incidence of cardiovascular events from 85 to 90 years of age. A second sensitivity analysis was done with risk groups according to site of CVD: a group with a history of cardiac CVD (angina pectoris and/or MI), a group with a history of cerebrovascular CVD (TIA and/or stroke), a group with a history of peripheral CVD (intermittent claudication and/or surgery for noncoronary arterial disease), and a group with a history of CVD at multiple sites. Data analysis was performed using SPSS 17.0 for Windows (SPSS Inc., Chicago, IL, USA).

## Results

### Baseline characteristics

For 570 of the 599 participants, all baseline measurements were available. At 85 years of age, 270 (47.4 %) participants had no history of CVD, 128 (22.4 %) participants had minor CVD, and 172 (30.2 %) participants had major CVD (Table 1). Participants with major CVD were more often men (47 versus 27 % for minor CVD and 28 % for no CVD, *p*_trend_ < 0.001) and more often institutionalized (23 versus 18 % for minor CVD and 15 % for no CVD, *p*_trend_ = 0.048). They had higher scores of disability (*p*_trend_ = 0.009) and their MMSE scores were the lowest (*p*_trend_ < 0.001). Only 36 % of the participants with minor CVD and 51 % of the participants with major CVD used aspirin or oral anticoagulants. Median systolic blood pressure was 154 mmHg (interquartile range [IQR], 143–166), median total cholesterol was 5.7 mmol/L (IQR, 4.9–6.4). Participants with major CVD had lower HDL cholesterol levels (*p*_trend_ < 0.001). Use of statins was minimal: no more than 1 % of all participants used lipid-lowering drugs. From all participants with heart failure (*n* = 74), more than half (*n* = 38 [51 %]) also had a history of major CVD.

**Table 1:** Baseline characteristics of participants from the Leiden 85-plus Study (*n* = 570), depending on cardiovascular history

	Total	CVD history			p for trend^a^
	570 (100)	None 270 (47.4)	Minor 128 (22.4)	Major 172 (30.2)	
Sociodemographic characteristics					
Women	379 (67)	195 (72)	93 (73)	91 (53)	<0.001
Net monthly income >750€	276 (49)	147 (55)	52 (41)	77 (45)	0.058
Post primary school education	197 (35)	104 (39)	41 (32)	52 (31)	0.091
Noninstitutionalized living	467 (82)	229 (85)	105 (82)	133 (77)	0.048
Functional status					
Cognitive function (MMSE)	26 (22–28)	27 (24–29)	26 (23–28)	25 (19–28)	<0.001
ADL disability	10 (9–15)	10 (9–13)	10 (9–15)	10 (9–16)	0.009
Subjective well-being (Cantril) ^b^	8 (7–9)	8 (6–9)	8 (7–8)	8 (7–9)	0.177
Depressive symptoms (GDS) ^b^	2 (1–3)	2 (1–3)	2 (1–4)	2 (1–3)	0.636
Cardiovascular characteristics					
Classic risk factors					
Hypertension ^c^	325 (58)	134 (50)	87 (68)	104 (64)	0.003
RR systolic, mmHg	154 (143–166)	155 (144–166)	154 (142–168)	153 (141–166)	0.332
Hypercholesterolemia ^d^	123 (22)	59 (22)	30 (24)	34 (22)	0.928
Total cholesterol, mmol/L	5.7 (4.9–6.4)	5.7 (5.0–6.4)	5.8 (4.9–6.4)	5.6 (4.8–6.3)	0.320
HDL cholesterol, mmol/L	1.3 (1.0–1.6)	1.3 (1.1–1.6)	1.3 (1.0–1.5)	1.1 (0.9–1.4)	<0.001
BMI, kg/m²	27 (24–30)	27 (25–30)	27 (24–31)	26 (24–29)	0.096
Diabetes ^e^	89 (16)	38 (14)	19 (15)	32 (20)	0.151
Smoking ^f^	267 (49)	122 (46)	54 (42)	91 (58)	0.029
Medication use					
Blood pressure lowering drugs ^g^	316 (57)	111 (42)	92 (72)	113 (71)	<0.001
Anticoagulants/aspirin	162 (28)	28 (10)	46 (36)	88 (51)	<0.001
Lipid-lowering drugs	6 (1)	1 (0.4)	2 (1.6)	3 (1.7)	0.15
Cardiovascular history					
Angina	109 (19)	0	60 (48)	49 (29)	
Transient ischemic attack	75 (13)	0	40 (31)	35 (21)	
Intermittent claudication	37 (7)	0	12 (10)	25 (15)	
Heart failure	74 (13)	0	36 (28)	38 (22)	
Myocardial infarction	99 (17)	0	0	99 (58)	
Stroke	61 (11)	0	0	61 (36)	
Surgery for arterial disease ^h^	42 (7)	0	0	42 (25)	

Data presented as n (%) for categorical variables, and median (IQR) for continuous variables

CVD = cardiovascular disease; No CVD = participants with no history of CVD; Minor CVD = participants with a history of angina, transient ischemic attack, intermittent claudication or heart failure; Major CVD = participants with a history of myocardial infarction, stroke or surgery for arterial disease ; MMSE = Mini-Mental State Examination (range 0-30); ADL = basic activities of daily living (range 9-36); Cantril = Cantril’s ladder of life (range 0–10); GDS = Geriatric Depression Scale (range 0–15)

^a^Chi-square test for categorical variables and Jonckheere-Terpstra for continuous variables;

^b^assessed only in participants with MMSE >18; ^c^ RR ≥160 systolic at baseline or a history of hypertension; ^d^ total cholesterol ≥6.5 at baseline or statin use; ^e^ history of diabetes, antidiabetic medication use or non-fasting glucose ≥11 mmol/L; ^f^ current or past smoker; ^g^ use of β-blockers, ACE inhibitors, diuretics or calciumchannel blockers as reported by the participants pharmacists

^h^aorta, carotid, coronary or peripheral arteries

At 90 years of age, 303 (53 %) participants were still alive. Follow-up for mortality was complete, and for 296 participants, we completed all clinical measurements at 90 years.

### Morbidity and mortality

**Fig. 1:**
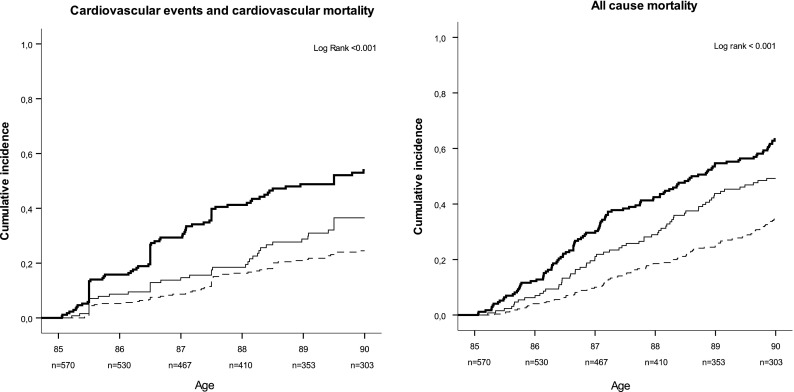
History of CVD and 5-year incidence of the composite endpoint “MI, stroke, and cardiovascular mortality” (*left panel*), as well as incidence of all-cause mortality (*right panel*) for three groups with no history of CVD, a history of minor CVD, and a history of major CVD, respectively. *Thick solid line* major CVD, *thin solid line* minor CVD, *dashed line* no CVD

During 5 years of follow-up, 181 (32 %) participants reached the composite endpoint, including 76 (42 %) fatal and nonfatal MI, 76 (42 %) fatal and nonfatal strokes, and 29 (16 %) additional cardiovascular deaths. Figure 1 shows the Kaplan–Meier curves for the three groups for the composite endpoint “incident cardiovascular events or cardiovascular mortality” (left panel) and all-cause mortality (right panel). Overall, during these 5 years, 267 (47 %) participants died; of which, 106 (40 %) died from cardiovascular causes. The incidence rate for “incident cardiovascular events or cardiovascular mortality” increased from 56 (95 % CI, 44–72) per 1,000 person-years at risk in the group with no CVD to 88 (95 % CI, 65–118) in the group with minor CVD and to 164 (95 % CI, 144–199) in the group with major CVD (Table 2). The risks for a fatal or nonfatal MI, a fatal or nonfatal stroke, and the composite endpoint increased from 1.7 (95 % CI, 0.9–3.1), 1.7 (95 % CI, 0.9–3.2), and 1.6 (95 % CI, 1.1–2.4), respectively, in participants with minor CVD to 2.6 (95 % CI, 1.6–4.5), 3.4 (95 % CI, 2.0–5.8), and 2.7 (95 % CI, 2.0–3.9), respectively, in those with major CVD. In a direct comparison of the group with major CVD with the group with minor CVD, the risk of the composite endpoint was nearly doubled in the major CVD group (HR, 1.8; 95 % CI, 1.2–2.7).

**Table 2:** Five-year hazard ratios and absolute risks of cardiovascular morbidity and mortality, depending on cardiovascular history adjusted for sex (*n* = 570)

			CVD history			*p* for trend	HR major versus minor
			None (*n* = 270)	Minor (*n* = 128)	Major (*n* = 172)		
Morbidity and mortality	Fatal and nonfatal MI	HR	1	1.7 (0.9–3.1)	2.6 (1.6–4.5)	0.001	1.8 (0.97–3.2)
		No. of events	25 (9.3)	17 (13.3)	34 (19.8)		
		Incidence rate	23 (15–33)	37 (23–58)	65 (47–90)		
	Fatal and nonfatal stroke	HR	1	1.7 (0.9–3.2)	3.4 (2.0–5.8)	<0.001	2.0 (1.1–3.6)
		No. of events	23 (8.5)	16 (12.5)	37 (21.5)		
		Incidence rate	20 (14–30)	35 (21–56)	69 (51–94)		
	CV events or CV mortality^a^	HR	1	1.6 (1.1–2.4)	2.7 (2.0–3.9)	<0.001	1.8 (1.2–2.7)
		No. of events	61 (22.6)	39 (30.5)	81 (47.1)		
		Incident rate	56 (44–72)	88 (65–118)	164 (144–199)		
Mortality	Cardiovascular	HR	1	2.0 (1.1–3.4)	3.7 (2.3–5.8)	<0.001	1.9 (1.2–3.1)
		No. of events	29 (10.7)	23 (18.0)	54 (31.4)		
		Incidence rate	25 (18–36)	48 (32–71)	95 (74–122)		
	Noncardiovascular	HR	1	1.5 (1.03–2.3)	1.7 (1.2–2.5)	0.001	1.1 (0.7–1.7)
		No. of events	66 (24.4)	40 (31.3)	55 (32.0)		
		Incidence rate	57 (54–72)	83 (62–111)	97 (75–124)		
	All-cause	HR	1	1.7 (1.2–2.3)	2.3 (1.7–3.1)	<0.001	1.4 (1.02–1.9)
		No. of events	95 (35.2)	63 (49.2)	109 (63.4)		
		Incidence rate	82 (68–100)	131 (104–164)	193 (162–227)		

Data are presented as HR with corresponding 95 % CIs, absolute numbers of events (%), and incidence rates as number of incidents per 1,000 person-years at risk with corresponding 95 % CIs

*CVD* cardiovascular disease, *No CVD* participants with no history of CVD, *Minor CVD* participants with a history of angina, TIA, intermittent claudication, or heart failure, *Major CVD* participants with a history of MI, stroke, or surgery for arterial disease (aorta, carotid, coronary, or peripheral arteries)

^a^Composite endpoint: fatal and nonfatal MI, fatal and nonfatal stroke, and cardiovascular mortality

For cardiovascular mortality, the risks increased from 2.0 (95 % CI, 1.1–3.4) in the minor CVD group to 3.7 (95 % CI, 2.3–5.8) in the major CVD group (*p*_trend_ < 0.001). For all-cause mortality, the risks rose from 1.7 (95 % CI, 1.2–2.3) in the minor group to 2.3 (95 % CI, 1.7–3.1) in the major group (*p*_trend_ < 0.001). After adjustment for the use of antihypertensive medication, income, and level of education, all these estimates remained roughly similar (data not shown).

When we analyzed the HRs with 10-year follow-up, we found similar risks for cardiovascular and all-cause mortality: HR, 1.5 (95 % CI, 0.99–2.2) and 1.4 (95 % CI, 1.1–1.8), respectively, for minor CVD and HR, 2.6 (95 % CI, 1.9–3.7) and 2.0 (95 % CI, 1.6–2.5), respectively, for major CVD.

### Functional status

**Fig. 2:**
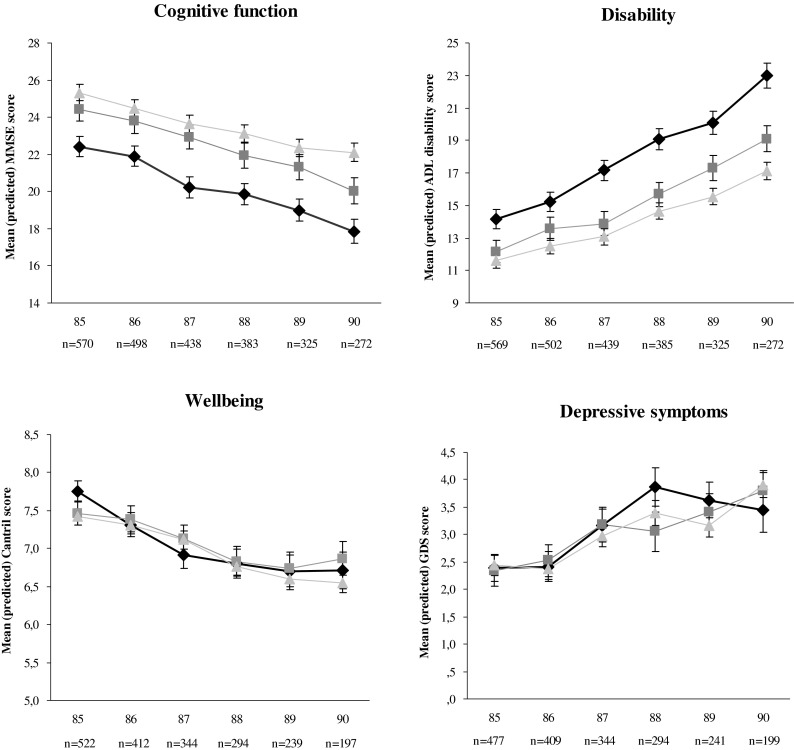
Changes in cognitive function, disability, subjective well-being, and depressive symptoms over time as estimated from linear mixed models adjusted for sex. Data are presented as the means with standard errors. *Triangles* reference group with no history of CVD, *squares* minor CVD, *diamonds* major CVD

At baseline, there were no differences in functional status between participants with minor CVD and those with no CVD (Table 3; Fig. 2). But the MMSE score was lower (−2.8 points, *p* < 0.001) and ADL disability score was higher (2.6 points, *p* = 0.003) in participants with major CVD. Compared to participants with no CVD, participants with minor CVD had an additional annual decrease in MMSE score of −0.19 points (*p* = 0.023) and increase in ADL disability score of 0.25 points (*p* = 0.042) over time. Participants with major CVD had an additional annual decrease in MMSE score (−0.24 points, *p* = 0.005) and increase in ADL disability score (0.61 points, *p* < 0.001). Compared to participants with minor CVD, participants with major CVD had an additional annual increase in ADL disability score of 0.36 points (*p* = 0.023). All other changes in functional status over time were not significant.

**Table 3:** Association between history of cardiovascular disease at 85 years of age and (changes in) functional status from 85 through 90 years of age (*n* = 570)

	Cross-sectional effect^a^				Annual effect reference group		Additional annual effect^a^			
	Minor CVD		Major CVD				Minor CVD		Major CVD	
	*B* (SE)	*p* value	*B* (SE)	*p* value	*B* (SE)	*p* value	*B* (SE)	*p* value	*B* (SE)	*p* value
MMSE	−0.65 (0.69)	0.35	−2.8 (0.69)	<0.001	−0.67 (0.045)	<0.001	−0.19 (0.084)	0.023	−0.24 (0.085)	0.005
ADL disability	0.58 (0.71)	0.41	2.6 (0.72)	0.003	1.1 (0.066)	<0.001	0.25 (0.12)	0.042	0.61 (0.13)	<0.001
Cantril scale of well-being	−0.021 (0.17)	0.90	0.24 (0.17)	0.15	−0.20 (0.021)	<0.001	0.039 (0.041)	0.35	−0.045 (0.043)	0.29
Geriatric depression scale	0.060 (0.31)	0.85	0.033 (0.31)	0.92	0.30 (0.035)	<0.001	−0.023 (0.069)	0.74	0.030 (0.070)	0.66

Associations were assessed by linear mixed models adjusted for sex. Scale: MMSE, 0–30 points; ADL disability, 9–36 points; Cantril, 0–10 points; GDS of depressive symptoms, 0–15 points

*CVD* cardiovascular disease

^a^Reference group: group with no CVD at baseline

### Sensitivity analyses

At 90 years of age, participants were recategorized with the updated clinical information from 85 to 90 years of age. Of the participants with complete data at 90 years of age (*n* = 296), 119 (40 %) had no history of CVD, 93 (31 %) had minor CVD, and 84 (28 %) had major CVD. In participants with minor CVD, the 5-year risk for cardiovascular mortality (up to 95 years of age) was not significantly increased (1.1; 95 % CI, 0.6–1.9), but participants with major CVD had a more than twofold increased risk (HR, 2.1; 95 % CI, 1.2–3.7). For all-cause mortality, the HRs were 1.1 (95 % CI, 0.79–1.5) and 2.1 (95 % CI, 1.5–3.0), respectively.

The second sensitivity analysis was done with different groups according to the site of their CVD. There were 25 participants (4.4 %) with peripheral CVD, 73 (12.8 %) with cerebrovascular CVD, 109 (19.1 %) with cardiac CVD, and 66 (11.6 %) with CVD on more than one site (Table 4). HRs were all calculated with the group with no CVD as reference group. The HR for fatal or nonfatal stroke was as high as 3.9 (95 % CI, 2.2–6.9) for those with previous TIA or stroke, and the HR for fatal or nonfatal MI was particularly high (3.4; 95 % CI, 1.9–6.4) in the group with CVD on multiple sites. The HR for cardiovascular mortality was highest in participants with peripheral CVD (3.8; 95 % CI, 1.7–8.5). In contrast with this high risk of cardiovascular mortality, the HRs for fatal or nonfatal MI and fatal or nonfatal stroke in participants with peripheral CVD were low, nearly equal to the group with no CVD (1.0; 95 % CI, 0.23–4.3 and 1.4; 95 % CI, 0.32–5.8, respectively). Except for the above-mentioned HRs, the groups did not differ materially.

**Table 4:** Five-year hazard ratios for cardiovascular morbidity and mortality for participants, depending on specific site of cardiovascular history adjusted for sex (*n* = 570)

		CVD history				
		None (*n* = 297)	Cardiac (*n* = 109)	Cerebrovascular (*n* = 73)	Peripheral (*n* = 25)	Multiple sites (*n* = 66)
Morbidity	Fatal and nonfatal MI	1	2.0 (1.1–3.7)	1.9 (0.96–3.9)	1.0 (0.23–4.3)	3.4 (1.9–6.4)
	Fatal and nonfatal stroke	1	2.3 (1.3–4.2)	3.9 (2.2–6.9)	1.4 (0.32–5.8)	1.6 (0.68–3.7)
	CV events or CV mortality^a^	1	2.0 (1.4–3.0)	2.3 (1.5–3.5)	1.9 (0.93–3.8)	2.5 (1.6–3.9)
Mortality	Cardiovascular	1	2.8 (1.7–4.6)	3.0 (1.7–5.3)	3.8 (1.7–8.5)	3.1 (1.7–5.5)
	Noncardiovascular	1	1.4 (0.90–2.1)	1.5 (0.95–2.4)	1.6 (0.76–3.3)	1.6 (1.0–2.6)
	All-cause	1	1.8 (1.3–2.5)	2.0 (1.4–2.8)	2.2 (1.3–3.8)	2.0 (1.4–3.0)

Data are presented as HR with corresponding 95 % CIs

*CVD* cardiovascular disease, *Cardiac* angina or MI, *Cerebrovascular* TIA or stroke, *Peripheral* claudication or operation for noncoronary arterial disease, *Multiple sites* CVD on more than one of cardiac, cerebrovascular, or peripheral sites

^a^Composite endpoint: fatal and nonfatal MI, fatal and nonfatal stroke, and cardiovascular mortality

## Discussion

In this cohort of oldest old from the general population, participants with a history of major CVD had a markedly increased risk of recurrent MI, stroke, and functional decline, as well as cardiovascular and all-cause mortality. Patients with a history of minor CVD had a relatively better prognosis: in nonagenarians, a history of minor CVD was not associated with mortality anymore. This implies that, within the group of very old patients who are eligible for secondary prevention, different risk groups can now be identified on the basis of clinical information only. Since the prevention of morbidity and subsequent loss of independency is increasingly important in the aging Western societies, cardiovascular prevention remains essential up to the highest age groups. Our findings underscore the importance of adequate secondary preventive measures in those with a history of major cardiovascular events, since these measures have been shown to be effective up to high ages (Andrawes et al. 2005; Alhusban and Fagan 2011; Castilla-Guerra et al. 2009; Asberg et al. 2010; Ray et al. 2006; Deedwania et al. 2007; Shepherd et al. 2002; Wenger and Lewis 2010; Thomas et al. 2010). On the other hand, our results suggest that the potential yield of secondary preventive measures in the oldest old might be less in older persons with a history of minor CVD.

Our study is the first to analyze prognosis based on a history of “minor” and “major” CVD in the general population of the oldest old. This distinction between minor and major CVD is based on the difference in risk of cardiovascular events and mortality after angina or TIA compared with the risk after an MI or stroke, which has been described in younger patients (Arima et al. 2006; Rosengren et al. 1998; Hjemdahl et al. 2006). Our study now also showed a significant difference in cardiovascular morbidity and mortality between groups with a history of minor and major CVD in participants aged 85 years and over.

Most other studies, performed in younger age groups, have evaluated risks after an event in a specific cardiovascular bed (cardiac, cerebral, or peripheral) (Migliaccio-Walle et al. 2010; Bhatt et al. 2009; Arima et al. 2006; Touze et al. 2005) or observed differences in risk between one or multiple CVD sites (Ferrieres et al. 2006; Migliaccio-Walle et al. 2010; Bhatt et al. 2009; Steg et al. 2007). The high risk of recurrent stroke in the group with a history of TIA or stroke that we observed is in line with previous studies (Steg et al. 2007; Arima et al. 2006; Ferrieres et al. 2006; Vickrey et al. 2002).

In keeping with earlier reports, in the present study, the risk of cardiovascular mortality was remarkably high in participants with peripheral artery disease, whereas cardiovascular morbidity risk in this group was relatively low (Ferrieres et al. 2006; Steg et al. 2007; Arima et al. 2006; Vickrey et al. 2002). In contrast with younger age groups, mortality risks in our study were not higher in participants with multiple-site CVD (Steg et al. 2007; Bhatt et al. 2009; Migliaccio-Walle et al. 2010; Ferrieres et al. 2006). In very old age, it seems that major CVD, rather than multiple CVD, is of prognostic value.

Previous studies have revealed a significant decline in physical functioning after stroke and MI in 70-year-olds (van Jaarsveld et al. 2001; Newman et al. 2009) and a negative impact on neurocognitive function (Waldstein and Wendell 2010; Newman et al. 2005, 2009). Our study confirmed these data in the very old.

Our study has several strengths. First, our results can be easily applied in day to day medical practice. The current electronic medical records provide clinicians a rapid overview of the cardiovascular history without any extra costs or effort. Secondly, our population-based study had a high participation rate (87 %) and no exclusion criteria, both allowing our conclusions to be generalized to the oldest old in the general population. Finally, we studied multiple relevant outcomes for an ageing population: mortality, morbidity, and functional status. A limitation might be that CVD history was based on the diagnosis of the primary care physicians, using variable diagnostic standards. However, this reflects clinical reality in primary care and previous research has shown the accuracy of data recorded in general practice to be very high (Hassey et al. 2001). In view of the fact that this study was the first to discriminate major and minor CVD in very old age, we recommend that this analysis be repeated in another cohort.

Evidence that medication for secondary cardiovascular prevention is recommendable up to the highest age groups is increasing (Andrawes et al. 2005; Alhusban and Fagan 2011; Castilla-Guerra et al. 2009; Asberg et al. 2010; Maroo et al. 2008; Ray et al. 2006; Deedwania et al. 2007; Shepherd et al. 2002; Wenger and Lewis 2010; Thomas et al. 2010). In this cohort, the use of secondary preventive medication was far from optimal, leaving room for improvement. From our results, it can be derived that distinct groups are discernible within those who should receive secondary prevention. This is an important finding, since at very old age, polypharmacy (Banerjee et al. 2011; Fleg et al. 2011), treatment adherence (Kvan et al. 2006), and the delicate balance between benefit and harm (Cardenas-Valladolid et al. 2010; Kvan et al. 2006; Fleg et al. 2011) raise a challenge for clinicians in day to day practice (Anthierens et al. 2010; Fried et al. 2011). The results of our study may help them to make appropriate treatment decisions, taking all relevant prognostic information into account.

In conclusion, in very old age, the CVD history is an easy tool for clinicians to identify patients who are at high risk for new cardiovascular events, functional decline, and cardiovascular mortality, as well as all-cause mortality. A history of major CVD nearly doubles the risk of a recurrent cardiovascular event or cardiovascular mortality compared with a history of minor CVD. Our results encourage both physicians and 85-year-olds with a history of major CVD to maximize their cardiovascular preventive efforts. However, when adverse effects or harmful interactions arise in a very old patient with minor CVD, the balance between benefit and harm could change and strict continuation of preventive medication might be reconsidered.

## Abbreviations

ADL: Activities of Daily Living
BNP: Brain natriuretic peptide
CABG: Coronary artery bypass grafting
CAD: Coronary artery disease
CI: Confidence interval
CV: Cardiovascular
CVD: Cardiovascular disease
GDS: Geriatric Depression Scale
HR: Hazard ratio
ICD: International Classification of Diseases
MI: Myocardial infarction
MMSE: Mini-Mental State Examination
PAD: Peripheral artery disease
PTCA: Percutaneous transluminal coronary angioplasty
SE: Standard error
TIA: Transient ischemic attack
